# COVID19‐Related Onset and Relapses of Juvenile Systemic Lupus Erythematosus‐Like Disease

**DOI:** 10.1111/jpc.70087

**Published:** 2025-05-22

**Authors:** Chiara Cannata, Francesca Tirelli, Alessandra Meneghel, Francesco Zulian

**Affiliations:** ^1^ Promise University of Palermo Palermo Italy; ^2^ Pediatric Rheumatology Unit, Department of Woman's and Child's Health University of Padova Padua Italy

**Keywords:** anakinra, case report, COVID‐19, juvenile systemic lupus erythematosus

**Summary**

- COVID‐19 pandemic has revealed the complex relationship between viral infection and the onset or exacerbation of autoimmune diseases such as systemic lupus erythematosus (SLE).
- In the more recent SARS‐CoV‐2 epidemics, most children present with mild symptoms or are asymptomatic, yet autoimmune manifestations have been reported.
- We report the case of a young girl who developed a SLE‐like disease following SARS‐CoV‐2 infection and two consecutive relapses, one following SARS‐CoV‐2 vaccination and another one after a COVID‐19 reinfection.

## 1 Case Report

A previously healthy 14‐year‐old female presented with fatigue, ecchymosis and menometrorrhagia. One month earlier, she had a flu‐like episode with fever and an upper airways infection for a few days.

Physical examination revealed malar rash, petechiae and ecchymosis, especially on lower limbs, without signs of any other internal organ involvement. Laboratory investigations showed severe hypochromic microcytic anaemia (haemoglobin 7 g/dL) with severe thrombocytopenia (platelet count 11.000/mm^3^), normal ESR, CRP, C3‐C4 complement fractions, coagulation profile and positive direct Coombs's test. Further immunological investigations showed the presence of antinuclear antibodies (ANA 1:640) and high titre extractable nuclear antigen (ENA) antibodies (anti‐RO/SSA), positive anti‐cardiolipin (IgM) and anti‐β2‐glycoprotein I antibodies (IgM). Lupus anti‐coagulant and anti‐dsDNA antibodies were negative. Serological analysis confirmed a recent Sars‐Cov‐2 infection (IgG 252 kBAU/L). Serum PCR and serology (IgG‐IgM) excluded a recent infection from common pathogens such as *Mycoplasma pneumoniae* , Adenovirus, Respiratory Syncytial Virus (RSV), Influenza A and B, Parvovirus B19, Epstein Barr virus (EBV) and cytomegalovirus (CMV).

According to the EULAR/ACR 2019 classification [1], she met systemic lupus erythematosus (SLE) criteria for ANA positivity, skin involvement, thrombocytopenia and antiphospholipid antibodies. She was treated with intravenous immunoglobulin (IVIG 1 g/kg), oral corticosteroids (CS) (prednisone 1 mg/kg/day) and hydroxychloroquine (HCQ) (200 mg/day), with a satisfactory clinical and haematological response. Both medications were gradually tapered down, allowing discontinuation of therapy after 5 months.

**FIGURE 1:**
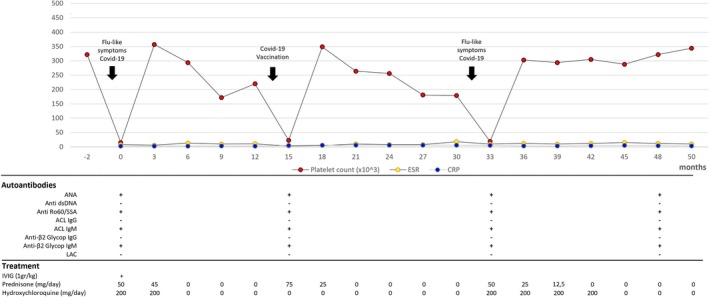
Timeline of the clinical case highlighting the progression of laboratory values and autoantibodies according to SARS‐CoV‐2 infections and vaccinations.

The patient remained asymptomatic off treatment for over 6 months but then, following SARS‐CoV‐2 vaccination, that we had advised, she experienced a disease relapse characterised by malar rash and thrombocytopenia (platelet count 16.000/mm^3^) (Figure 1).

Given the onset of disease approximately 1 month after a COVID‐19 infection, confirmed by positive serology for SARS‐CoV‐2 and the exacerbation after vaccination, a possible COVID‐19‐related SLE‐like disease (CR‐jSLE) was suspected. A subsequent 3‐month course of CS was effective, as evidenced by clinical and laboratory remission. The patient remained well, off treatment, for a further 14 months; then, 1 month after a new febrile flu‐like episode, which affected the entire family group, she presented a new relapse of thrombocytopenia (platelet count 19.000/mm^3^) with an increased anti‐Sars‐Cov2 antibody titre (IgG 808 kBAU/L). A new 6‐month course of CS and HCQ treatment was started and clinical remission was achieved. At 50 months since the disease onset and 12 months off therapy, she is clinically well and has not experienced new relapses (Figure 1).

## 2 Discussion

It is well known that SLE onset can be triggered by various infectious agents such as Epstein–Barr virus (EBV), Cytomegalovirus (CMV) and Retrovirus [2, 3] but little is known about the role of Sars‐Cov2 virus in this context, especially in children. SARS‐CoV‐2 virus, which caused the global COVID‐19 pandemic, has not only challenged healthcare systems worldwide but also revealed complex interactions with autoimmune phenomena. Well documented as a potent inducer of autoimmune responses, SARS‐CoV‐2 virus has been implicated in both the initiation and exacerbation of autoimmune conditions during the acute phase of the disease and its aftermath [4]. The young patient, herein described, developed a SLE‐like syndrome following SARS‐CoV‐2 infection and two consecutive relapses, one following SARS‐CoV‐2 vaccination and another one after a proven re‐infection. Despite some typical symptoms and the immunological features, the absence of anti‐dsDNA, normal ESR, C3 and C4 complement fractions, the rapid and effective response to brief courses of corticosteroids, followed by a sustained remission without immunosuppressive treatment and lack of renal involvement, quite frequent in the paediatric age, were unusual for conventional jSLE [5]. It is well known that worldwide autoimmune conditions seem to have increased since the onset of the COVID pandemic. These findings, along with clinical and serological evidence of recent SARS‐CoV‐2 infection, led us to hypothesise a COVID‐19‐related condition characterised by a favourable response to less aggressive treatment and good prognosis.

To date, only two other paediatric cases with proven CR‐jSLE‐like characteristics have been reported [6, 7]. The clinical history and laboratory features of these patients and ours are summarised in Table 1.

**TABLE 1:** Summary of Covid19‐related juvenile SLE‐like disease cases.

Author, year (references)	Age	Sex	Previous Covid‐19 history	Clinical picture	Laboratory	Treatment	SLE classification criteria [1]	Long‐term outcome
Asseri 2022 [6]	13	F	Proven disease 2 months earlier	DAH, Renal involvement	Hemolytic anaemia, ANA+, dsDNA+, low C3‐C4	Cs, PE, MMF, HCQ	ANA + score 15	N/A
De Belo 2022 [7]	11	F	Proven disease 1 month earlier	Arthritis, Serositis	ANA+, dsDNA+, ENA+ (histone, nucleosoma) low C3	CS, HCQ	ANA + score 17	Off‐therapy at 12 months follow up
Present case	15	F	Probable disease 1 month earlier, positive serology	Thrombo cytopenia, Skin rash	ANA+, ENA+ (anti‐RO/SSA), anti‐cardiolipin+ (IgM), anti‐β2‐glycoprotein+ (IgM)	CS, HCQ	ANA + score 10	Off‐therapy at 12 months follow up

Abbreviations: ANA, antinuclear antibodies; CS, corticosteroids; DAH, diffuse alveolar haemorrhage; dsDNA, anti‐double strain DNA; ENA, extractable nuclear antigens; HCQ, hydroxychloroquine; MMF, mofetil mycophenolate; PE, plasma exchange.

Patients were all female teenagers with no significant past medical history. In all, COVID‐19 preceded the onset of symptoms by 1–2 months. All of them presented severe illness requiring hospital admission and fulfilled the revised 2019 EULAR/ACR classification criteria for SLE as for systemic clinical manifestations and immunological characteristics [1]. All patients underwent corticosteroids and HCQ treatment while mofetil mycophenolate (MMF) and plasma exchange (PE) were needed in one patient with diffuse alveolar haemorrhage (DAH) (Table 1). All patients achieved disease remission that was maintained for more than 12 months in two of them. In the third patient, presenting with DAH, the long‐term follow up was not reported [6].

The rapid recovery observed in these patients further suggests that CR‐jSLE may represent a distinct clinical entity, requiring an individualised approach to management and follow‐up. Compared to the other two cases, the distinctive feature in our patient was the relapsing disease course following either a new episode of SARS‐CoV‐2 infection or vaccination. The latter event provides an interesting insight into the complex interplay between vaccination and autoimmunity. Although SARS‐CoV‐2 vaccines rarely cause serious adverse events, cases of new‐onset autoimmune diseases, such as immune thrombotic thrombocytopenia, autoimmune liver diseases, Guillain‐Barré syndrome, IgA glomerulonephritis and SLE, have been reported [8]. Molecular mimicry and activation of toll‐like receptors on antigen‐presenting cells have been postulated to play a role in the development of vaccine‐induced autoimmune diseases [8, 9]. Kanduc et al. [9] found that the SARS‐Cov2 spike protein shares similarities with human proteins at the heptapeptide level, making the molecular mimicry between human proteins and the spike protein antigen plausible.

The recurrence of symptoms after a subsequent febrile illness in our patient also raises the possibility of a “*hit and run*” mechanism, where the immune system remains hyper‐responsive long after the initial viral insult. This scenario is consistent with findings suggesting increased relapse rates of autoimmune manifestations following additional viral exposures after COVID‐19 [4]. A recent study in adults has confirmed the association between COVID‐19 and autoimmune diseases such as rheumatoid arthritis (adjusted hazard ratio (aHR) 2.98), SLE (aHR 2.99), vasculitis (aHR 1.96), as well as inflammatory bowel disease (aHR 1.78) and type‐1 diabetes mellitus (aHR 2.68) [10].

In conclusion, this long‐term follow‐up of a case study provides quite reliable evidence that the SARS‐CoV‐2 virus may be linked to the onset or relapse of a less typical and more indolent form of jSLE (CR‐jSLE). This underscores the critical importance of personalised treatment strategies, i.e., just a short course of CS avoiding heavy immunosuppression, and close monitoring of this subset of patients, particularly in the context of new SARS‐CoV‐2 infections or vaccination. A larger epidemiological study is on‐going to confirm this preliminary observation.

## Ethics Statement

According to the Padua University Hospital policy, approval from the Ethics Committee was not needed because all information was anonymously collected.

## Consent

The patient provided written informed consent.

## Conflicts of Interest

The authors declare no conflicts of interest.
